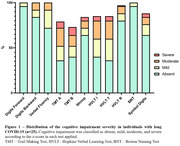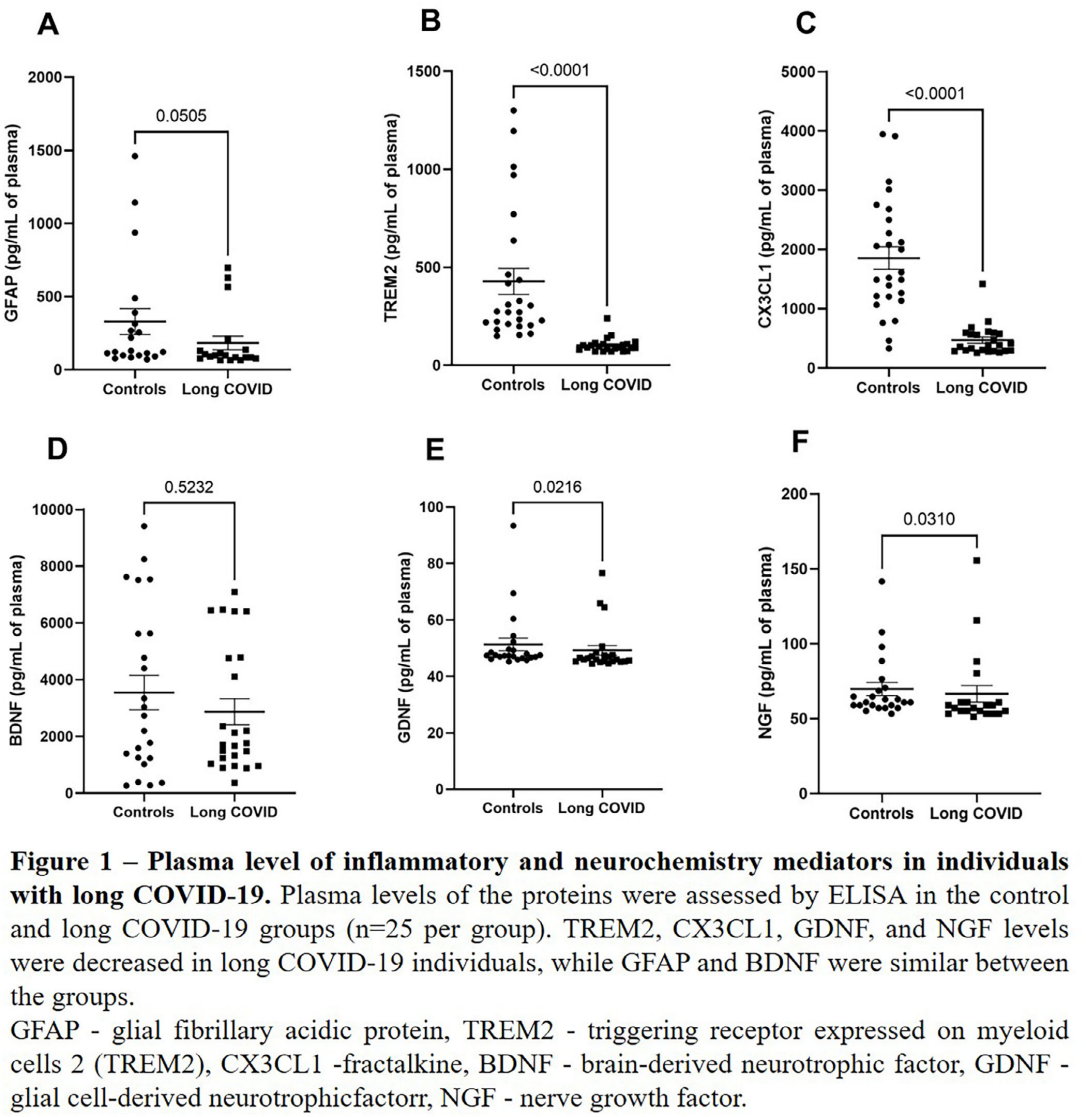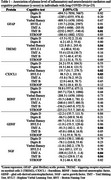# Plasma levels of inflammatory and neurochemical mediators are associated with cognitive impairment in long COVID‐19

**DOI:** 10.1002/alz70855_107215

**Published:** 2025-12-24

**Authors:** Nadia Shigaeff, Celso Martins Queiroz‐Júnior, Thiago Guilerme Rêgo Barros, Eugenio Damaceno Hottz, Aline Silva Miranda, Vivian Vasconcelos Costa, Eliana Cristina de Brito Toscano

**Affiliations:** ^1^ Federal University of Juiz de Fora, Juiz de Fora, Minas Gerais, Brazil; ^2^ Federal University of Minas Gerais, Belo Horizonte, na, Brazil; ^3^ Federal University of Juiz de Fora, Juiz de Fora, Brazil; ^4^ Federal University of Minas Gerais, Belo Horizonte, Brazil; ^5^ Federal University of Juiz de Fora, Medical School, Juiz de Fora, Minas Gerais, Brazil

## Abstract

**Background:**

COVID‐19 is associated with clinically significant symptoms despite the resolution of the acute infection, characterizing a post‐COVID‐19 syndrome or long COVID‐19. Cognitive impairment is among the most common of these symptoms. Recent evidence has demonstrated a close correlation between COVID‐19 and neurodegeneration, bringing the potential role of COVID‐19 in the future development of dementia into the spotlight. In this scenario, neuroinflammation is emerging as a potential mechanism linking these disorders. We aimed to evaluate the cognitive function and the plasma levels of inflammatory and neurochemistry mediators in individuals with long COVID‐19 compared with age‐ and sex‐matched healthy controls (*n* = 25 per group) younger than 65 years. We also sought to investigate the possible association between these proteins and cognitive impairment.

**Method:**

Plasma levels of GFAP, TREM2, CX3CL1, BDNF, GDNF, and NGF were quantified using immunoenzimatic assay (ELISA). Cognitive function was evaluated through a comprehensive neuropsychological battery, comprising digits forward and backward, verbal fluency (animals), Trail Making Test (TMT), Stroop, Hopkins Verbal Learning Test (HVLT), Boston Name Test, and symbol digits. We applied the Mann‐Whitney test to compare control with long COVID‐19 group, and linear regression to examine the association of plasma proteins with cognitive functions.

**Result:**

Both groups had 23 women (92%), and the median age of the controls was 39 (19‐52) and 35.1 (21‐59) in the long COVID‐19 group (*p* = 0.12). The post‐infecction interval was 8.2 (6‐12) months. Long COVID‐19 individuals had a more severe impairment in the TMT, HVLT, and Stroop tests (Figure 1). In addition, they presented decreased plasma levels of TREM2, CX3CL1, GDNF, and NGF compared with healthy controls (Figure 2). Worse performance in TMT was associated with increased levels of GFAP, GDNF, and NGF; while increased levels of TREM2 and CX3CL1 were associated with better performance in the HVTL (imediated) test. Patients with increased TREM2 levels also got higher scores on the verbal fluency test (Table 1).

**Conclusion:**

Changes in glial activity and expression of neurotrophic factors are associated with long COVID‐19. Plasma levels of neurochemical mediators may be useful in predicting impairment of frontal cognitive functions.